# Metal-Modified Biochar Activates Persulfate for the Removal of Phenolic Pollutants from Water: Mechanism Prediction and Non-Radical Targeted Regulation

**DOI:** 10.3390/toxics14050409

**Published:** 2026-05-09

**Authors:** Wenxuan Wei, Wenqian Cao, Ruijuan Qu, Zunyao Wang

**Affiliations:** State Key Laboratory of Water Pollution Control and Green Resource Recycling, School of the Environment, Nanjing University, Nanjing 210023, China; weiwenxuan_edu@163.com (W.W.); 13199531169@163.com (W.C.); quruijuan0404@nju.edu.cn (R.Q.)

**Keywords:** phenolic pollutants, persulfate oxidation technology, metal-modified biochar, non-radical, machine learning

## Abstract

Phenolic pollutants are toxic and widespread, making their removal an urgent priority. Metal-modified biochar (MBC) shows great potential for non-radical activation of persulfate (PS), enabling efficient and low-carbon removal of phenolic pollutants. However, regulating the non-radical mechanism remains challenging due to the lack of systematic understanding of how metal-modification-related preparation parameters influence the reaction pathway. Herein, a machine learning (ML) framework focusing on metal modification parameters was introduced. Five ML algorithms were used to build binary classification models. XGBoost achieved the best performance, with an area under the receiver operating characteristic curve (AUC) of 0.711 on the independent test set and both precision and recall of 0.733 for identifying the non-radical-dominated mechanism. SHapley Additive exPlanations (SHAP) and partial dependence analysis reveal that high temperatures (>800 °C) and moderate heat treatment time (1.7–2.5 h) favor non-radical pathways, with Cu introduction and multi-metal synergy serving as key regulatory factors. Based on SHAP dependency analysis and metal combination statistics, two strategies for the targeted design of non-radical-dominated systems are proposed: (1) prepare Cu-modified biochar at 900 °C; (2) prepare Cu-Fe bimetallic-modified biochar at 600 °C. This work provides a data-driven theoretical framework and operational strategies for targeted regulation of the non-radical mechanism in the MBC/PS system, opening new avenues for the efficient and low-carbon treatment of phenol-containing wastewater.

## 1. Introduction

Phenolic compounds are a typical class of toxic organic pollutants. These pollutants are widely present in wastewater from industries such as petrochemicals, coal mining, papermaking, and pharmaceuticals, within a concentration range of 0.1–3900 mg/L [1,2,3]. Phenolic pollutants can enter the body through skin or mucous membrane contact, causing protein denaturation and cellular damage, and also disrupting the endocrine system, thereby posing a serious threat to human health and ecosystems [4,5,6]. Consequently, the development of efficient and cost-effective treatment technologies for phenol-containing wastewater has become a research focus in the environmental field [7].

Persulfate (PS)-based oxidation technology has shown broad application prospects in the treatment of phenolic pollutants due to its advantages of simple operation, wide pH applicability, and strong oxidizing capacity [8,9]. PS can generate radical or non-radical active species through heterogeneous activation [10]. Among these, the non-radical pathway exhibits higher oxidant utilization efficiency, selectivity, and resistance to interference [9,11]. Therefore, the development of non-radical-dominated PS systems is of great significance for the efficient and low-carbon treatment of phenol-containing wastewater. Biochar, as a low-cost, easily prepared, and environmentally friendly carbon material, has attracted widespread attention in the field of PS heterogeneous catalysis [12,13]. Metal-modified biochar (MBC), obtained through metal-loading modification, combines the high catalytic activity of metals with the adsorption and electron-transfer advantages of biochar, exhibiting excellent performance in the non-radical activation of PS [14,15,16]. Although numerous studies have reported various MBC-based systems for the non-radical activation of PS to remove phenolic pollutants from water, current research has primarily focused on the performance evaluation and mechanistic elucidation of individual catalysts, lacking macro-level exploration and quantitative understanding of how multiple factors influence the reaction mechanism.

In recent years, the rise of machine learning (ML) technology has provided a new data-driven paradigm for the rational design of catalysts [17,18,19]. By uncovering hidden patterns in large amounts of experimental data, ML can accelerate the development of PS systems and the targeted regulation of their mechanisms [20,21,22]. However, existing studies have mostly focused on modification strategies such as the selection of biochar precursors, the control of thermal treatment parameters, and heteroatom doping [23,24,25]. Metal modification, as the most common biochar modification method, is often simplified or overlooked in data-driven modeling.

To address these research gaps, this study incorporates the characteristics of metal modification into data-driven modeling and constructs a binary classification prediction model to identify the dominant reaction mechanism of MBC/PS systems. The contribution and influence patterns of various factors on the reaction mechanism are quantitatively evaluated, providing guidance for the targeted design of non-radical-dominated systems. The main contents include: (1) systematically collecting literature data to construct a database of MBC/PS systems for the removal of phenolic pollutants; (2) employing various ML algorithms to establish a binary classification prediction model for reaction-dominant mechanisms, and determining the optimal model through hyperparameter optimization and multi-dimensional evaluation metrics; (3) combining the SHAP method with partial dependency analysis to reveal the key factors influencing non-radical mechanisms and their influence patterns; (4) proposing a directed design strategy for non-radical mechanisms in MBC. This study provides data support and theoretical guidance for the targeted regulation of the MBC/PS system, and offers a new technological pathway for the efficient, low-carbon treatment of phenolic pollutants in water.

## 2. Materials and Methods

### 2.1. Literature Screening

To visually illustrate the systematic construction of the dataset and the overall analytical pipeline, a comprehensive flowchart encompassing literature screening, data collection, data processing, and model development is provided in Figure S1.

All data in the dataset were extracted from published literature. The literature was sourced from the Science Citation Index Expanded (SCI-E) in the Web of Science Core Collection to ensure that the data originated from reliable studies. The search terms “persulfate OR peroxydisulfate OR peroxymonosulfate (Topic) and biochar (Topic)” were used to cover all studies related to biochar-activated persulfate systems. The search period was set from 2021 to 2025 (publication year), and a total of 1797 research papers were retrieved.

To ensure that the dataset focuses on the system of MBC-activated PS for removing phenolic pollutants from water, this study developed an automated text cleaning and filtering program for titles and abstracts using the Python programming language and its Pandas data processing library. The specific filtering logic is as follows:

1. Text preprocessing and standardization.

First, the “Title” and “Abstract” fields from each literature entry were concatenated into a single string and uniformly converted to lowercase. This step eliminates interference caused by differences in capitalization formats across sources during retrieval matching, thereby forming a unified global search text source.

2. Inclusion of the target system.

To define the scope of this study, the inclusion logic requires that the text contain relevant terms for “PS” (including persulfate, peroxydisulfate, and peroxymonosulfate), “biochar”, and “phenolic pollutants”. To avoid missing literature that uses abbreviations or common names for phenolic pollutants, this study constructed a multidimensional phenolic feature dictionary. The dictionary mainly includes: (1) core roots: phenol and cresol, broadly covering various conventional phenolic compounds; (2) classic model pollutants: polyhydroxy benzenes such as catechol and hydroquinone; (3) novel endocrine disruptors containing phenolic rings: steroidal estrogens such as estradiol and estrone; (4) specific phenol-containing drugs and antimicrobial agents: acetaminophen and triclosan; (5) frequently used abbreviations: BPA (bisphenol A), PCP (pentachlorophenol), 4-NP (4-nitrophenol), and other common abbreviations.

3. Exclusion of interfering systems

To eliminate interference from other physical and chemical mechanisms, activation systems involving external energy input (e.g., literature explicitly containing keywords such as photocatalysis, electrocatalysis, thermal activation, UV light, ultrasound, and microwave-assisted processes), non-PS systems (e.g., ozone oxidation, Fenton/hydrogen peroxide systems), and non-water pollution control systems (e.g., soil remediation) were excluded.

Through this automated screening process, a total of 224 articles highly relevant to the system of “biochar and its derivatives activating PS to remove phenolic pollutants from water” were identified. Since text-matching algorithms cannot comprehensively cover all descriptive forms of metal modification, a manual review was conducted to further select 109 articles from the aforementioned 224 articles, focusing on “MBC-activated PS for the removal of phenolic pollutants from water”. From these 109 articles, a total of 132 independent data points were extracted. Multiple entries from a single article were permitted, provided there were definitive variations in the core feature variables. The data used in this study and their source articles are detailed in Table S1.

### 2.2. Data Collection

To control feature dimensionality and ensure model stability on a limited dataset, this study treats diverse phenolic pollutants as a unified target group and focuses primarily on catalyst-related characteristics. Based on the results of the literature review and practical research experience, this study identified 19 feature variables: (1) heat treatment parameters: preparation route (Preparation_Route), maximum preparation temperature (Max_Temp), and duration at the maximum preparation temperature (Max_Time); (2) metal introduction: introduction of metal elements (Fe, Co, Ni, Cu, Mn, Al, Zn, Mg, Mo, Ce, Bi, Ca) and the number of metal elements (Metal_Count); (3) material physicochemical properties: specific surface area (BET_Surface_Area) and the intensity ratio of the D band to the G band in the Raman spectrum (Id_Ig_Ratio); (4) reaction system: oxidant type (Oxidant_Type). The definition, assignment method, and data type of each feature variable are detailed in Table 1.

In PS-AOP systems, radical (e.g., SO_4_^•^^−^, •OH and O_2_^•−^) and non-radical pathways often coexist. The contribution of non-radical pathways (*R*_N_) was calculated using Equation (1), and the radical contribution is given by 1 − *R*_N_.

(1) $$R_{N}=\frac{\eta_{\mathrm{total}}-\eta_{\mathrm{scar}}-\eta_{\mathrm{ads}}}{\eta_{\mathrm{total}}-\eta_{\mathrm{ads}}},$$

*η*_total_ (%)—total pollutant removal efficiency without a radical quencher;

*η*_ads_ (%)—adsorption removal rate with only catalyst (no oxidant);

*η*_scav_ (%)—the removal rate attributed specifically to radical species. This value is obtained by selectively summing the removal decreases caused by multiple quenchers (e.g., methanol or ethanol for SO_4_^•−^ and •OH, tert-butanol for •OH, and p-benzoquinone for O_2_^•−^), carefully avoiding double-counting of overlapping scavenger effects.

In binary classification, a default probability threshold of 0.5 is typically used to assign class labels [26,27]. Therefore, a system with *R*_N_ ≥ 0.5 was defined as non-radical-dominated (Non-radical = 1), and *R*_N_ < 0.5 as radical-dominated (Non-radical = 0). It is crucial to emphasize that this 0.5 cutoff serves as an operational and statistical threshold for machine learning modeling, rather than a strict mechanistic boundary. In practical MBC/PS systems, radical and non-radical pathways typically coexist to varying degrees. Thus, the binary labels herein represent the dominant mechanism rather than mutually exclusive pathways. The boxplot of *R*_N_ (Figure S2) shows a median of 0.543 and an interquartile range of [0.215, 0.898] for the full dataset (*N* = 132), indicating a relatively balanced distribution and supporting the rationality of the 0.5 threshold. To account for potential literature-induced label noise and verify the stability of this classification, a sensitivity analysis utilizing alternative thresholds (*R*_N_ = 0.4 and 0.6) was conducted. Through this classification, complex kinetic data were converted into standardized binary target labels, enabling classification algorithms to identify the core factors driving qualitative changes in the reaction mechanism.

All data in this dataset were sourced from explicit descriptions or numerical values in the main text or Supplementary Materials of the literature. For data presented only as images, the WebPlotDigitizer-4.7 software was used for numerical extraction to ensure the accuracy of the collected data.

### 2.3. Data Preprocessing

To ensure the generalization capability and rigor of the ML model, this study adopted a strict “split-first, then process” strategy to prevent data leakage. After converting the collected 132 sets of experimental data into a formatted dataset, stratified random sampling was performed at the sample level to split the dataset into a training set (*N* = 105) and a test set (*N* = 27) at an 8:2 ratio. Continuous variables were winsorized at the 1st and 99th percentiles using the training set as the reference. Specifically, values below the 1st percentile were set to the 1st percentile value, and values above the 99th percentile were set to the 99th percentile value. Features with a missing rate exceeding 0.300 were excluded [28]. For features with a missing rate not exceeding 0.300, missing values were imputed using the K-nearest neighbor (KNN) algorithm (*K* = 5). This algorithm identifies the five known samples most similar to the missing sample in the multidimensional feature space and calculates the missing value using distance-weighted averaging. Subsequently, data were standardized using the Z-score method.

All parameters for the interpolation and standardization rules were derived solely from the training set and then applied to the test set using the same transformations. The test set was not used for any parameter estimation.

### 2.4. Feature Selection

The intergroup discriminatory ability of each feature variable was evaluated as follows. Samples were divided into two groups based on the dominant reaction mechanism: the radical-dominated system group (Non-radical = 0) and the non-radical-dominated system group (Non-radical = 1). For continuous variables, an independent samples *t*-test was used to compare the mean differences between the two groups. For binary variables, a chi-square test was used to compare the frequency distributions.

The Pearson correlation coefficient (*r*) was used to assess multicollinearity between feature variables. |*r*| < 0.4 indicates weak correlation; 0.4 ≤ |*r*| < 0.6 indicates moderate correlation; |*r*| ≥ 0.6 indicates strong correlation.

L1-regularized logistic regression (L1-LR) was employed for sparse feature weighting. The model used the dominant mechanism (non-radical vs. radical) as the binary target variable, and all candidate variables retained in the previous steps served as input features. The regularization strength is controlled by the parameter *C* (*C* = 1/λ, where λ is the regularization coefficient). Using 5-fold cross-validation (5-CV) and the area under the receiver operating characteristic curve (AUC) as the performance metric, the value of *C* was searched across a predefined grid (generated in logarithmic space). The *C* value that yielded the maximum cross-validation AUC was selected as the optimal regularization parameter. At the optimal *C* value, the regression coefficient of each feature was recorded. A positive coefficient indicates a positive contribution to the non-radical pathway; a negative coefficient indicates a negative contribution; a coefficient of zero indicates that the feature was eliminated during the sparsification process.

### 2.5. Model Construction and Evaluation

To identify classification models with optimal fitting and generalization capabilities, this study selected four mainstream ensemble tree models—eXtreme Gradient Boosting (XGBoost), Light Gradient Boosting Machine (LightGBM), Categorical Boosting (CatBoost), and Random Forest (RF)—to construct binary classification models for predicting the dominant reaction mechanisms. The linear model L1–LR (described in Section 2.4) was also evaluated as a baseline. Detailed descriptions of the models are provided in Text S1.

Model performance was evaluated using the confusion matrix, AUC, the approximate area under the precision–recall curve (AP), the Brier score of the calibration curve, and decision curve analysis (DCA). The calculation methods for these evaluation metrics are described in Text S2. Bootstrap resampling and the DeLong test were used to assess the statistical significance of the AUC differences between models.

### 2.6. Model Interpretability Methods

To reveal the marginal contribution and influence direction of each feature on the prediction results, this study employed the SHapley Additive exPlanations (SHAP) algorithm for model interpretability analysis. Based on the Shapley value from game theory, SHAP calculates the average marginal contribution of each feature across all possible feature subsets, yielding an attribution value (SHAP value) for each feature in every sample. A positive SHAP value indicates that the feature positively contributes to predicting the non-radical pathway, while a negative value indicates a negative contribution. The absolute value reflects the strength of the influence. SHAP values for each feature were calculated on the training set.

To characterize the nonlinear marginal effects between key features and the prediction target, this study employed a combination of partial dependence plots (PDP) and individual conditional expectation (ICE) plots. PDP calculates the average model prediction by fixing the target feature to a specific value in the training set (while keeping other features at their original values), thereby displaying the average marginal effect of that feature. ICE plots the predicted value as a function of the target feature for each individual sample, reflecting heterogeneity at the individual level.

To identify the optimal single-metal and bimetallic combinations, the proportion of non-radical samples under different metal introduction conditions was statistically analyzed based on the training set. For single-metal cases, the proportion of non-radical mechanisms was calculated using samples containing only that metal (with no other metals present). For bimetallic combinations, the proportion was calculated using samples containing both specified metals (and no other metals).

### 2.7. Computing Environment and Implementation

All data preprocessing, modeling, evaluation, and visualization were performed using Python 3.7.16 and its scientific computing and machine learning libraries, primarily including Pandas (1.3.5), NumPy (1.21.5), Scikit-learn (1.0.2), XGBoost (1.6.2), LightGBM (4.6.0), CatBoost (1.0.6), SHAP (0.42.1), Matplotlib (3.5.3), Seaborn (0.12.2), and pauc (0.1.7). Some statistical analyses were performed using SciPy (1.7.3) and Statsmodels (0.13.5).

## 3. Results and Discussion

### 3.1. Analysis of the Dataset

Table S2 shows the missing data rates for the initial 19 feature variables. A total of 94.7% of the feature variables had missing rates not exceeding 0.300, indicating that the dataset is relatively complete. The missing rates for Max_Temp, Max_Time, BET_Surface_Area, and Id_Ig_Ratio were 0.008, 0.015, 0.212, and 0.333, respectively. The remaining feature variables had no missing values. The KNN interpolation algorithm is a common method for imputing missing values [29]. For features with a missing rate not exceeding 0.300, this study employed the KNN algorithm (*K* = 5) to impute missing values. To ensure dataset quality, the feature variable Id_Ig_Ratio, which had a missing rate exceeding 0.300, was excluded [30].

Descriptive statistical analysis was performed on the training set to clarify the overall data distribution and preliminarily evaluate the discriminatory power of each feature variable across groups. As shown in Table 2, the sample sizes of the non-radical-dominated system group (Non-radical = 1, *N* = 59) and the radical-dominated system group (Non-radical = 0, *N* = 46) are relatively balanced, laying a solid data foundation for the stable training of subsequent ML models.

Regarding heat treatment parameters, the mean maximum preparation temperature (Max_Temp) for the non-radical group was 728.81 ± 144.20 °C, slightly higher than that of the radical group (695.43 ± 165.20 °C). The specific surface area (BET_Surface_Area) of the non-radical group was also higher. More intense heat treatment promotes graphitization of the biochar carbon framework, enhancing material conductivity. A higher specific surface area provides more adsorption sites for oxidants and pollutants, thereby facilitating non-radical pathways such as high-valent metal (HVM) and electron transfer pathways (ETP) [31]. Regarding transition metal dopants, the data reveal distinct mechanistic tendencies. The frequency of Fe in the radical group (80.4%) is higher than that in the non-radical group (67.8%). This may be due to traditional iron-based materials readily inducing homogeneous Fenton-like reactions dominated by radical pathways [32]. Conversely, the proportions of Cu and Mn in the non-radical group (22.0% and 18.6%) are higher than those in the radical group (10.9% and 10.9%), suggesting that Cu and Mn have the potential to induce non-radical mechanisms. Furthermore, the mean number of metal types (Metal_Count) in the non-radical group (1.39 ± 0.59) is higher than that in the radical group (1.24 ± 0.43). This suggests that multi-metal synergy may facilitate the transition of the material mechanism from radical-based to non-radical-based pathways.

To identify feature variables with significant differences between the two groups, this study conducted univariate tests for between-group differences. As shown in Table 2, from a traditional univariate perspective, the *p*-values for all features examined were greater than 0.05. This indicates that the mechanism of the MBC/PS system cannot be determined by a single feature but is rather the result of nonlinear coupling and synergistic effects of multidimensional features.

During the data cleaning stage, the overall proportion of non-zero samples for eight elements (Ni, Al, Zn, Mg, Mo, Ce, Bi, and Ca) in the training set was less than 5%. Such extremely sparse features cannot provide effective signals. They are prone to introducing random noise and increase the risk of overfitting. Therefore, they were excluded [33].

**Figure 1:**
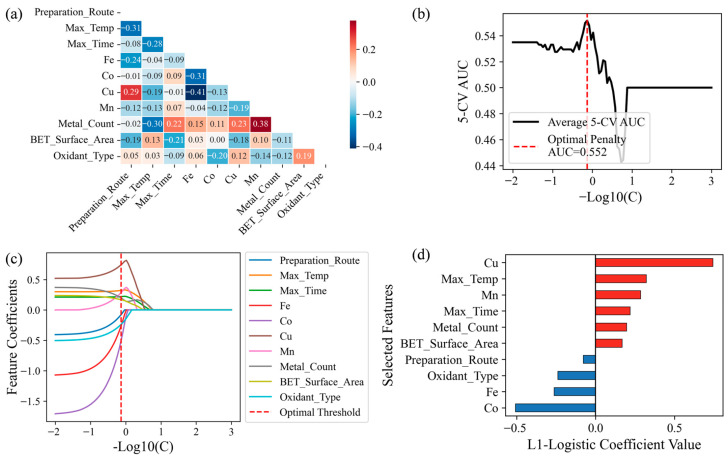
(**a**) Pearson correlation heatmap of feature variables. (**b**) Average 5-CV AUC versus regularization parameter for L1-LR. (**c**) Shrinkage path of feature variable coefficients. (**d**) Standardized coefficients of feature variables in the L1-LR model.

To assess the degree of redundancy among feature variables and mitigate the potential interference of multicollinearity on model interpretability, this study calculated the Pearson correlation coefficient matrix for the remaining 10 feature variables (Figure 1a). The results show that the absolute correlation coefficients (|*r*|) for the vast majority of feature variable pairs are less than 0.4, indicating weak correlation. A small number of variable pairs exhibit moderate correlation (0.4 < |*r*| ≤ 0.6), maintaining an overall acceptable level of multicollinearity.

L1-LR was used to compress feature weights and facilitate linear interpretation. Figure 1b shows the average 5-CV AUC as a function of the regularization parameter. At the optimal parameter (−log10(*C*) = −0.131), the validation set AUC reached its peak (0.552). Although the discriminatory power of this linear model is limited, all 10 candidate features maintained non-zero coefficients at this threshold (Figure 1c). This indicates a certain linear association with the reaction mechanism and suggests that these features are worthy of further investigation.

The feature coefficients determined based on the optimal hyperparameters are shown in Figure 1d. The positive and negative distributions in the bar chart reveal the direction of each variable’s marginal contribution to the formation of non-radical pathways. Cu exhibits a positive regression coefficient, suggesting that it plays an important role in driving the non-radical activation of PS. Conversely, the coefficients for Fe and Co are both negative. Both Max_Temp and BET_Surface_Area show positive contributions to the non-radical pathway. This aligns with the theoretical understanding that increased graphitization of carbon materials and a well-developed pore structure facilitate electron transfer or surface complexation mechanisms. The characteristic variable Metal_Count, representing multi-component doping, exhibits a positive coefficient, indicating the potential value of multi-metal synergistic sites in mechanism regulation.

Overall, L1-LR, as a linear screening tool, identified 10 features with non-zero coefficients, indicating that they have a linear association with the reaction mechanism. However, the model’s average 5-CV AUC was only 0.552, suggesting that linear models cannot adequately capture the complex relationship between material properties and reaction pathways. Given that this relationship is often highly nonlinear, more sophisticated ensemble tree models were employed in subsequent studies for in-depth prediction and mechanism analysis.

### 3.2. Construction and Evaluation of Machine Learning Prediction Models

**Figure 2:**
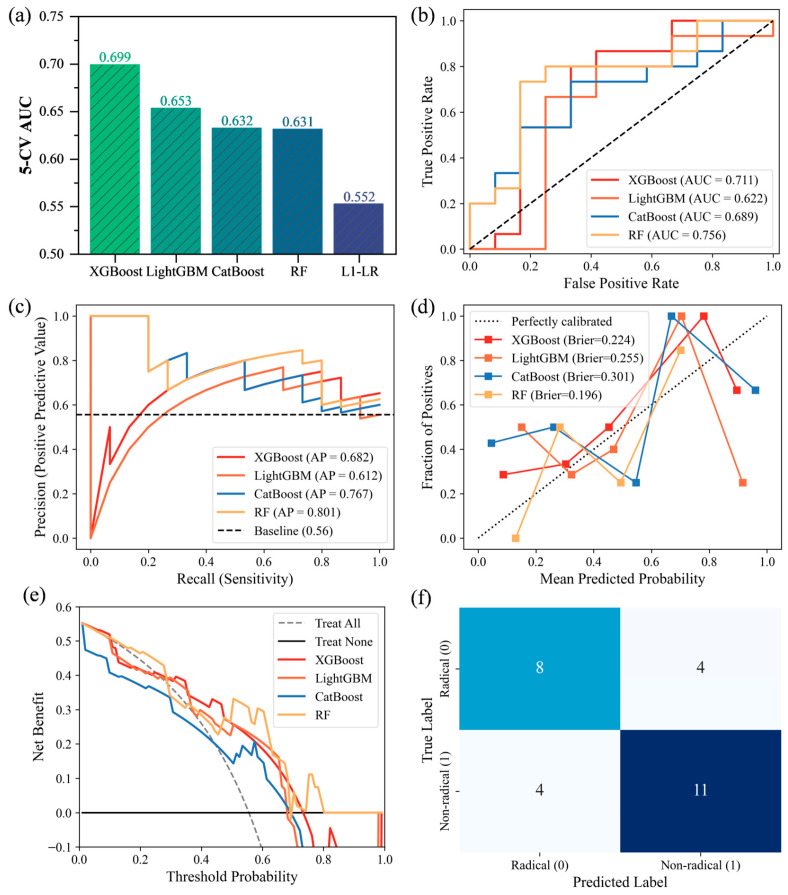
(**a**) Average 5-CV AUC of each model with optimal hyperparameters on the training set. (**b**) ROC curves, (**c**) P-R curves, (**d**) calibration curves, and (**e**) DCA on the independent test set. (**f**) Confusion matrix of XGBoost on the test set.

To identify classification models with optimal fitting and generalization capabilities, this study selected four mainstream ensemble tree models (XGBoost, LightGBM, CatBoost, and RF). These models were used to construct binary classifiers for predicting the dominant reaction mechanism, and their performance was evaluated. Model generalization was ensured by employing 5-CV combined with grid search for hyperparameter tuning, using AUC as the performance metric. The optimal hyperparameters for each model on the training set are shown in Table S3. As shown in Figure 2a, all tree-based nonlinear ensemble models outperformed the linear model L1-LR (AUC = 0.552). Under 5-CV, the average AUC values were 0.699 for XGBoost, 0.653 for LightGBM, 0.632 for CatBoost, and 0.631 for RF. This indicates a nonlinear coupling relationship between the preparation parameters and physicochemical properties of metal-modified biochar and the reaction mechanism, rather than a simple linear superposition.

To comprehensively evaluate the generalization performance of each model on unseen data, this study used an independent test set comprising 20% of the total sample size (*N* = 27) to systematically evaluate the models across multiple dimensions, including receiver operating characteristic (ROC) curves, precision–recall (P-R) curves, calibration curves, and DCA.

As shown in Figure 2b,c, on the independent test set, the RF model achieved the highest AUC (0.756) and AP (0.801). XGBoost (AUC = 0.711, AP = 0.682) and CatBoost (AUC = 0.689, AP = 0.767) performed slightly worse than RF but still demonstrated good generalization capabilities. LightGBM (AUC = 0.622, AP = 0.612) performed the worst among the four ensemble tree models. In the evaluation of probability prediction reliability, the calibration curves (Figure 2d) show that the Brier scores for the XGBoost and RF models are 0.224 and 0.196, respectively, both below 0.25. This indicates good consistency between their predicted probabilities and the true frequencies [34]. As shown in Figure 2e, DCA further confirms that the XGBoost model consistently provides stable and significant positive net gains across a wide range of threshold probabilities. This suggests that using this model to guide decision-making for the targeted synthesis of non-radical materials will significantly outperform traditional empirical trial-and-error methods [35].

Notably, the AUC of RF on the independent test set (0.756) was considerably higher than its average 5-CV AUC on the training set (0.631). Given the small sample size of the test set (*N* = 27), this elevated AUC may reflect chance bias in sample distribution rather than true generalization ability. Bootstrap analysis yielded a 95% confidence interval (CI) of [−0.247, 0.129] for the AUC difference between XGBoost and RF, which includes zero, and the DeLong test gave a *p*-value of 0.638 (>0.05) [36]. These results indicate no statistically significant difference between the two models, indicating they can be considered comparable candidates. However, XGBoost achieved the highest average 5-CV AUC on the larger training set (*N* = 105) (0.699), consistent with its performance on the test set (0.711), suggesting lower sensitivity to fluctuations in data distribution. To further evaluate robustness and eliminate single-split bias, 100 repetitions of stratified 5-CV were performed. Across these 500 independent evaluations, XGBoost again yielded a higher mean AUC (0.611) than RF (0.565), confirming its superior discriminative ability across diverse data splits (Figure S3). Therefore, XGBoost was selected for further evaluation.

The classification confusion matrix of XGBoost on the independent test set (Figure 2f) and the classification report (Table 3) show that the model achieved an overall classification accuracy of 0.704 across 27 samples. For the core prediction target “Non-radical mechanism (Non-radical = 1)”, the model correctly identified 11 out of 15 true non-radical samples (recall = 0.733), with precision and F1-score both 0.733. This indicates that the XGBoost model demonstrates good balance and accuracy in identifying non-radical pathways.

In summary, the XGBoost model not only demonstrated the strongest fitting capability on the training set but also maintained high accuracy and good generalization performance on the independent test set. Therefore, this study selected the optimized XGBoost as the benchmark model for subsequent mechanism identification and feature importance analysis to identify key drivers of reaction pathways and elucidate the intrinsic relationships between material properties and reaction mechanisms.

### 3.3. Model Interpretation and Strategy Development

#### 3.3.1. Feature Influence Analysis

Model interpretability is a key issue in machine learning research. Although ensemble tree models exhibit excellent predictive performance, their internal decision-making processes are often regarded as a “black box” with limited interpretability [17]. This study employed a game theory-based interpretability framework using the SHAP algorithm to quantitatively analyze the marginal contributions of each feature. Combined with PDP to characterize the nonlinear relationships between key features and the prediction target, the study revealed the importance of each feature variable in the XGBoost model built on the MBC/PS dataset, as well as their influence patterns on the reaction mechanism.

**Figure 3:**
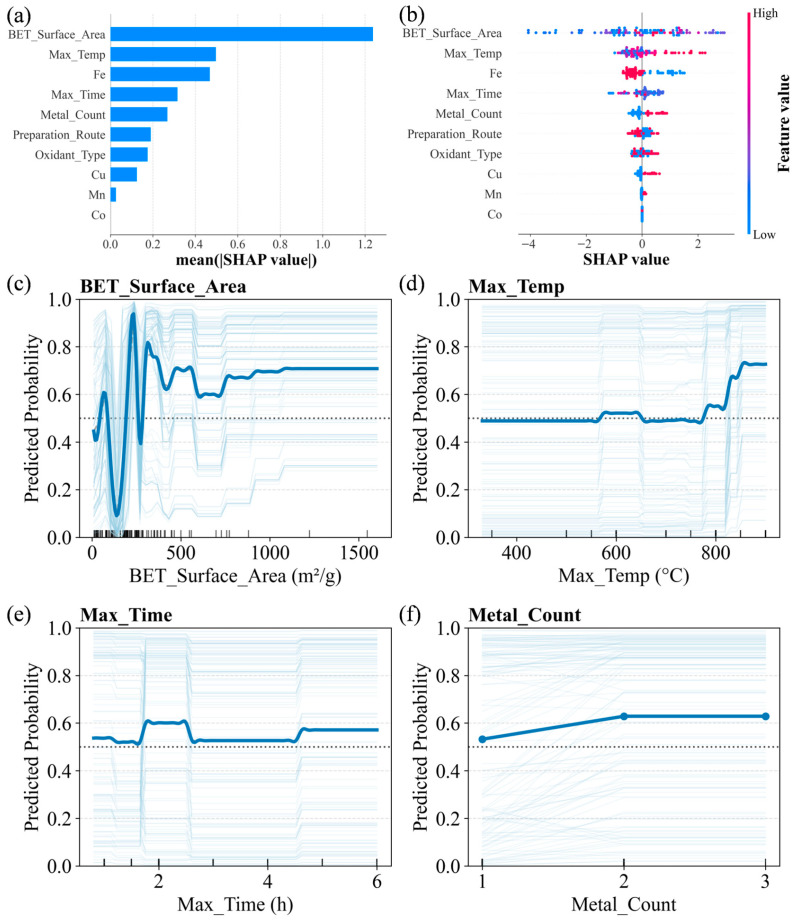
SHAP summary plots: (**a**) feature importance bar plot and (**b**) beeswarm plot. PDP-ICE analysis for key feature variables: (**c**) BET_Surface_Area, (**d**) Max_Temp, (**e**) Max_Time, and (**f**) Metal_Count. Light blue lines represent ICE curves, and dark blue lines represent the PDP curves.

The impact of each feature variable on the model predictions was quantified by averaging their absolute SHAP values calculated on the training set. As shown in Figure 3a, BET_Surface_Area has the greatest influence on predicting the reaction mechanism, suggesting that in heterogeneous catalytic systems, the surface pore structure of the catalytic material significantly affects the reaction pathway. Max_Temp and Max_Time also exhibit high SHAP values, attributed to their critical roles in determining the graphitization degree of carbon materials and the evolution of active sites. The introduction of Fe and Metal_Count rank as the third and fifth most important features, respectively. This indicates that, given a certain physical structure, the intrinsic properties of transition metal active centers and the synergistic configuration of multiple metals are key factors in regulating the reaction mechanism.

Furthermore, variations in experimental setups across different source literature introduce inherent label noise into the calculation of the *R*_N_ target variable. To address this uncertainty and verify model robustness, a sensitivity analysis was conducted using alternative classification thresholds (*R*_N_ = 0.4 and 0.6). As detailed in Table S4, the XGBoost model maintained acceptable predictive performance (For *R*_N_ = 0.4: training 5-CV AUC = 0.708, test AUC = 0.665; For *R*_N_ = 0.6: training 5-CV AUC = 0.617, test AUC = 0.698). Crucially, the top five most influential features (BET_Surface_Area, Max_Temp, Fe, Metal_Count and Max_Time) remained generally consistent in SHAP feature importance rankings across different thresholds (Figure S4). This stability confirms that the primary mechanistic inferences derived from the model are highly robust to literature-induced variability.

Figure 3b illustrates the influence patterns of feature variable values on model outputs. For Max_Temp, high values (red) correspond to SHAP values concentrated on the right side of the zero axis, indicating a significant positive contribution to non-radical mechanisms. Previous studies have shown that as the pyrolysis temperature of biochar increases, the number of persistent radical active sites that favor radical mechanisms gradually decreases. In contrast, the surface adsorption capacity and electron conduction capacity, which favor non-radical mechanisms, gradually increase. This drives the biochar/PS system to shift its dominant mechanism from radical-dominated to ETP-dominated non-radical pathways [37]. The type and number of metal elements significantly influence the orientation of the reaction mechanism. SHAP values for Fe-modified biochar systems are mainly distributed to the left of the zero axis, indicating a positive contribution to the radical pathway. This is because iron active sites readily induce homogeneous Fenton-like reactions, generating SO_4_^•−^ [38,39]. Additionally, when Fe coordinates with the weak-field ligand PMS, it adopts a high-spin state, and its unpaired electron can break the O−O bond via a single-electron pathway to generate radicals [10]. In contrast, the introduction of Cu exhibits a positive SHAP contribution. Cu’s lower spin makes it less likely to break O−O bonds via a single-electron pathway to generate radicals. Multiple studies have shown that CuO can activate PMS or PDS through outer-sphere interactions, forming metastable intermediates that remove pollutants via ETP [40,41,42,43]. The introduction of Mn and Co contributes little to SHAP and has no significant effect. Notably, systems with a higher number of metal species (Metal_Count) exhibit a significant positive SHAP contribution, indicating that bimetallic or multimetallic synergistic effects are an effective strategy for driving the transition of single-metal-modified biochar systems from radical pathways to non-radical pathways. However, it must be emphasized that these SHAP-derived insights should be considered a mechanistic hypothesis rather than direct evidence. Future experimental studies utilizing electron paramagnetic resonance (EPR) spectroscopy or radical quenching tests are necessary to explicitly validate these ML-inferred mechanisms.

A combination of PDP and ICE plots (Figure 3c–f) to quantify the marginal effects of key feature variables was employed on model predictions. The ICE curves reveal the nonlinear relationship between the target feature and the prediction results by holding other variables constant for individual samples, while the PDP curves reflect the average response across all samples [44].

As shown in Figure 3c, when BET_Surface_Area is below 400 m^2^/g, the predicted probability fluctuates significantly with increasing specific surface area. However, the overall upward trend of the PDP curve still indicates that an increase in specific surface area positively promotes the non-radical pathway. As shown in Figure 3d, Max_Temp exhibits a positive correlation with the probability of non-radical mechanism formation. In the range of 200–800 °C, the response values remain relatively stable, with a local peak near 600 °C. When the temperature exceeds 800 °C, the predicted probability undergoes a significant jump. This phenomenon can be attributed to a sudden change in the graphitization degree of carbon-based materials [37]. Figure 3e illustrates that the influence of Max_Time on the predicted probability of non-radical mechanisms exhibits a non-monotonic, multi-stage characteristic, with an optimal window (1.7–2.5 h) for the non-radical pathway. This indicates that a moderate heat treatment time can effectively promote the non-radical pathway, whereas times that are too short or too long are detrimental to its formation. As shown in Figure 3f, the PDP curve for Metal_Count exhibits an increasing trend as the number of metal types increases. When the number increases from 1 to 2, the predicted probability of the non-radical pathway shows a significant increase, reflecting the potential of bimetallic or multimetallic synergistic sites to overcome the limitations of single-metal mechanisms.

Based on SHAP feature analysis and PDP-ICE marginal effects from the XGBoost model, this study quantitatively reveals how key material preparation parameters, metal introduction, and physicochemical properties independently influence reaction pathway selection in the MBC/PS system. The results indicate that high-temperature heat treatment (>800 °C) and moderate heat treatment duration (1.7–2.5 h) favor the induction of non-radical pathways. The introduction of Cu and the construction of bimetallic/multimetallic synergistic sites are key strategies for achieving targeted regulation of non-radical pathways. However, further quantitative analysis is needed to elucidate the synergistic regulatory mechanisms between specific preparation parameters and metal introduction.

#### 3.3.2. Targeted Design Strategies for MBC

**Figure 4:**
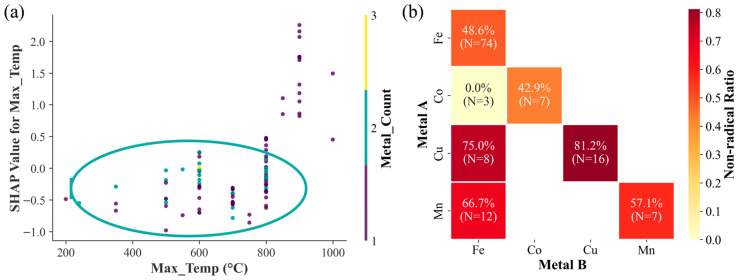
(**a**) SHAP dependence plot of the interaction between Max_Temp and Metal_Count. (**b**) Heatmap of synergistic effects for metal combinations.

The preceding analysis indicates that higher synthesis temperature (Max_Temp) and greater number of metal species (Metal_Count) are key factors for inducing non-radical mechanisms in the MBC/PS system. To further explore the synergistic interaction between these two factors, a SHAP dependence plot was constructed (Figure 4a). The results show that for single-metal modification (Metal_Count = 1), a synthesis temperature of 900 °C yields a high positive non-radical contribution. For dual/multi-metal modification (Metal_Count ≥ 2), the SHAP value peaks at 600 °C. This indicates that for single-metal modification, a temperature of 900 °C provides a significant positive benefit, whereas for dual/multi-metal modification, a temperature of 600 °C achieves the optimal benefit.

To screen for optimal metal combinations, the proportion of non-radical samples under different metal combinations was calculated. In Figure 4b, “Metal A” and “Metal B” represent the transition metals introduced into the MBC. Following the definitions in Section 2.6, the diagonal cells represent single-metal systems, while the off-diagonal cells denote bimetallic combinations. The results show that the Cu single-metal system had the highest proportion of non-radical samples (81.2%, *N* = 16, 95% CI: 57.0–93.4%), making it the single metal with the greatest potential for non-radical induction. Among the bimetallic combinations, the non-radical sample proportions for Co-Fe, Cu-Fe, and Mn-Fe were 0.0% (*N* = 3, 95% CI: 0.0–56.1%), 75.0% (*N* = 8, 95% CI: 40.9–92.9%), and 66.7% (*N* = 12, 95% CI: 39.1–86.2%), respectively. The Cu-Fe combination had the highest non-radical proportion and was therefore identified as the optimal bimetallic combination.

Based on the above results, two strategies for achieving targeted regulation of non-radical pathways in the MBC/PS system are proposed: (1) prepare Cu-modified biochar at a synthesis temperature of 900 °C; (2) prepare Cu-Fe bimetallic-modified biochar at a synthesis temperature of 600 °C.

## 4. Conclusions

In this study, a machine learning model was developed to predict the dominant reaction mechanisms in a system where MBC activates PS to remove phenolic pollutants from water. The study quantitatively revealed the influence of key features on non-radical pathways and proposed strategies for targeted catalyst design. The key findings are as follows: (1) The reaction mechanism is determined by the nonlinear coupling of multidimensional features, and no single variable can independently distinguish it (*p* > 0.05). (2) The XGBoost model demonstrated the best performance, with an AUC of 0.711 on the independent test set and precision and recall of 0.733 for the non-radical category. (3) High temperature (>800 °C), moderate heat treatment duration (1.7–2.5 h), Cu doping, and multi-metal synergy are key factors inducing non-radical pathways. (4) Two synthesis strategies are proposed: Cu monometal modification requires 900 °C, while Cu-Fe bimetallic modification achieves optimal results at 600 °C.

These findings provide data-driven support for the rational design of catalysts for non-radical degradation of phenolic pollutants. However, it is important to note that the relatively small sample size (*N* = 132) poses an inherent risk of overfitting, despite the rigorous cross-validation employed. The predictive capability of the current model is limited to interpolation within the specific parameter boundaries of the collected literature. Extrapolation beyond these dataset limits should be approached with caution. Furthermore, since this study treats diverse phenolic compounds as a unified target group, the specific dependence of reaction mechanisms on pollutant molecular structures remains to be fully explored. Future research incorporating high-throughput experiments could expand the dataset scale to enhance the model’s generalization ability, minimize literature-induced label noise, and integrate molecular descriptors of target pollutants. This would further elucidate the synergistic mechanisms of multiple features and the dynamic regulation of reaction pathways.

## Figures and Tables

**Table 1:** List of initial feature variables.

Category	Feature Variable Name	Meaning, Units, and Assignment Notes	Variable Type
Heat treatment parameters	Preparation_Route	Preparation route category. 0: One-step method (co-pyrolysis, one-step hydrothermal method, etc.) 1: Two-step method (carbonization followed by metal introduction via calcination, hydrothermal treatment, or ball milling)	Classification
	Max_Temp	Maximum preparation temperature (°C)	Continuous
	Max_Time	Processing time at maximum preparation temperature (h)	Continuous
Metal introduction	Fe, Co, Ni, Cu, Mn, Al, Zn, Mg, Mo, Ce, Bi, Ca	Metal element introduction status. 1: Introduced; 0: Not introduced	Classification
	Metal_Count	Number of metal component types introduced. 1: Single metal, 2: Two metals, etc.	Discrete
Material physicochemical properties	BET_Surface_Area	Specific surface area of the material (m^2^/g)	Continuous
	Id_Ig_Ratio	Intensity ratio of the D band to the G band in Raman spectroscopy; reflects the defect degree of carbon materials	Continuous
Reaction system	Oxidant_Type	Oxidant type. 0: Peroxymonosulfate (PMS) 1: Peroxydisulfate (PDS)	Classification
Dominant mechanism	Non-radical	Target variable. Whether the system is dominated by non-radical mechanisms. 1: Yes; 0: No	Classification

**Table 2:** Training set sample statistics.

Feature Variable Name	Non-Radical = 0 (*N* = 46)	Non-Radical = 1 (*N* = 59)	*p*-Value
Preparation_Route	26 (56.5%)	28 (47.5%)	0.468
Max_Temp	695.43 ± 165.20	728.81 ± 144.20	0.281
Max_Time	2.15 ± 1.18	2.33 ± 1.31	0.465
Fe	37 (80.4%)	40 (67.8%)	0.218
Co	5 (10.9%)	3 (5.1%)	0.461
Cu	5 (10.9%)	13 (22.0%)	0.213
Mn	5 (10.9%)	11 (18.6%)	0.409
Ni	1 (2.2%)	3 (5.1%)	0.795
Al	2 (4.3%)	2 (3.4%)	1.000
Zn	1 (2.2%)	2 (3.4%)	1.000
Mg	0 (0.0%)	2 (3.4%)	0.588
Mo	1 (2.2%)	2 (3.4%)	1.000
Ce	0 (0.0%)	0 (0.0%)	1.000
Bi	0 (0.0%)	1 (1.7%)	1.000
Ca	0 (0.0%)	1 (1.7%)	1.000
Metal_Count	1.24 ± 0.43	1.39 ± 0.59	0.133
BET_Surface_Area	273.48 ± 288.09	314.28 ± 326.77	0.499
Oxidant_Type	24 (52.2%)	26 (44.1%)	0.530

**Table 3:** Classification report of XGBoost on the test set.

	Precision	Recall	F1-Score	Number of Samples
0	0.667	0.667	0.667	12
1	0.733	0.733	0.733	15
Accuracy	/	/	0.704	27
Macro average	0.700	0.700	0.700	27
Weighted average	0.704	0.704	0.704	27

## Data Availability

The raw data supporting the conclusions of this article will be made available by the authors on request.

## Supplementary Materials

The following supporting information can be downloaded at: https://www.mdpi.com/article/10.3390/toxics14050409/s1. Text S1: Introduction of the models; Text S2: Calculation methods for the evaluation metrics; Figure S1: Comprehensive flowchart of the entire ML procedure; Figure S2: Boxplot of the contribution of non-radical pathways (*R*_N_) in the full dataset; Figure S3: Distribution of AUC across 100 repetitions of stratified 5-CV for each model; Figure S4: SHAP feature importance rankings under thresholds of (a) *R*_N_ = 0.4 and (b) *R*_N_ = 0.6; Table S1: List of metal-modified biochar (MBC) catalysts and their corresponding Web of Science accession numbers (UT); Table S2: Missingness and imputation methods of feature variables in the dataset; Table S3: Average 5-CV AUC and optimal hyperparameters of each model; Table S4: Predictive performance of XGBoost model under various thresholds.

## Abbreviations

The following abbreviations are used in this manuscript:

MBC	Metal-modified biochar
PS	Persulfate
AUC	Area under the receiver operating characteristic curve
SCI-E	Science Citation Index Expanded
KNN	K-nearest neighbor
L1-LR	L1-regularized logistic regression
5-CV	5-fold cross-validation
XGBoost	eXtreme Gradient Boosting
LightGBM	Light Gradient Boosting Machine
CatBoost	Categorical Boosting
RF	Random Forest
AP	Area under the precision–recall curve
DCA	Decision curve analysis
SHAP	SHapley Additive exPlanations
PDP	Partial dependence plots
ICE	Individual conditional expectation
HVM	High-valent metal
ETP	Electron transfer pathways
ROC	Receiver operating characteristic
P-R	Precision–recall
CI	Confidence interval
EPR	Electron paramagnetic resonance
