# Supplementary material for: Metal-Modified Biochar Activates Persulfate for the Removal of Phenolic Pollutants from Water: Mechanism Prediction and Non-Radical Targeted Regulation
Source: Toxics. 2026 May 9;14(5):409. doi: 10.3390/toxics14050409 (PMC13211714; doi:10.3390/toxics14050409)
Supplement: Supplementary file 1 [file toxics-14-00409-s001.zip › toxics-4281070-supplementary.pdf]

Supplementary Material

Metal-modified biochar activates persulfate for the removal of phenolic pollutants from water: Mechanism prediction and non-radical targeted regulation

Wenxuan Wei [1], Wenqian Cao [1], Ruijuan Qu [1] and Zunyao Wang [1,*]

[1] State Key Laboratory of Water Pollution Control and Green Resource Recycling, School of the Environment, Nanjing University, Jiangsu Nanjing 210023, PR China; weiwenxuan_edu@163.com (W.W.); 13199531169@163.com (W.C.); quruijuan0404@nju.edu.cn (R.Q.); wangzy@nju.edu.cn (Z.W.)

* Correspondence: wangzy@nju.edu.cn

Text S1. Introduction of the models.

Small datasets are prone to overfitting by machine learning (ML) algorithms, which may lead to significant generalization errors on test data. To minimize this risk, five models were used to train the dataset and identify the optimal model: L1-regularized logistic regression (L1-LR), eXtreme Gradient Boosting (XGBoost), Light Gradient Boosting Machine (LightGBM), Categorical Boosting (CatBoost), and Random Forest (RF).

L1-LR is a linear classifier that incorporates an L1 penalty to enforce sparsity in feature weights, enabling simultaneous feature selection and classification. This property makes it useful for high-dimensional or noisy datasets by automatically identifying the most relevant predictors while maintaining the interpretability of standard logistic regression. However, like other linear models, its ability to capture complex nonlinear relationships among features is limited. XGBoost, LightGBM, and CatBoost are optimized implementations of gradient boosting decision trees. By iteratively fitting the residuals of the previous model, they progressively reduce prediction bias, offering significant advantages in fitting complex nonlinear relationships and handling high-dimensional features. As a representative algorithm of the Bagging strategy in ensemble learning, RF constructs multiple decision trees and averages their predictions, demonstrating strong resistance to overfitting and robustness to noisy data.

Text S2. Calculation methods for the evaluation metrics.

A confusion matrix is a tabular tool used to evaluate the performance of classification models. By comparing the predicted class with the actual class, it visually displays the model's classification results across different categories. For binary classification problems, the confusion matrix contains four basic elements:

True positives (TP): the number of samples that are actually positive and correctly predicted as positive.

True negatives (TN): the number of samples that are actually negative and correctly predicted as negative.

False positives (FP): the number of samples that are actually negative but incorrectly predicted as positive (Type I errors).

False negatives (FN): the number of samples that are actually positive but incorrectly predicted as negative (Type II errors).

The confusion matrix presents these four metrics in tabular form, typically with the actual class as rows and the predicted class as columns, providing a visual representation of correct and incorrect classifications. Based on the confusion matrix, this study calculates the following core evaluation metrics:

Accuracy: The proportion of all samples that were correctly classified, reflecting the model's overall correctness (Eq. S1).

$$\text{Accuracy}=\frac{\text{TP+TN}}{\text{TP+TN+FP+FN}} \quad \text{(Eq. S1)}$$

Precision: The proportion of samples predicted as positive that are actually positive. It measures the reliability of the model's positive predictions (Eq. S2).

$$\text{Precision}=\frac{\text{TP}}{\text{TP+FP}} \quad \text{(Eq. S2)}$$

Recall: The proportion of actually positive samples that the model correctly identifies. It measures the model's sensitivity to the positive class (also known as sensitivity or true positive rate) (Eq. S3).

$$\text{Recall}=\frac{\text{TP}}{\text{TP+FN}} \quad \text{(Eq. S3)}$$

F1-Score: The harmonic mean of precision and recall, comprehensively reflecting the balance between precision and sensitivity (Eq. S4).

$$\text{F1}=2\times\frac{\text{Precision}\times\text{Recall}}{\text{Precision}+\text{Recall}} \quad \text{(Eq. S4)}$$

The classification report outputs precision, recall, and F1-score separately for each class, along with summary statistics such as macro average and weighted average to comprehensively evaluate the model's performance across all classes.

The receiver operating characteristic (ROC) curve is constructed by plotting the true positive rate (TPR) against the false positive rate (FPR) at all possible classification thresholds. For each threshold, samples are classified as positive or negative based on the predicted probability, and TPR (Eq. S5) and FPR (Eq. S6) are calculated from the confusion matrix.

$$\text{TPR}=\frac{\text{TP}}{\text{TP+FN}} \quad \text{(Eq. S5)}$$

$$\text{FPR}=\frac{\text{FP}}{\text{FP+TN}} \quad \text{(Eq. S6)}$$

Area under the ROC curve (AUC) quantifies the area under the ROC curve and represents the probability that a randomly selected positive sample has a higher predicted

score than a randomly selected negative sample. It is calculated using the trapezoidal rule, summing the areas of trapezoidal segments under the curve. AUC ranges from 0 to 1, with higher values indicating better discriminatory ability. The ROC curve and AUC are insensitive to class imbalance, making them suitable for imbalanced datasets.

The precision-recall (P-R) curve is constructed by plotting precision against recall at all possible classification thresholds. For each threshold, precision and recall are calculated based on the confusion matrix. Average precision (AP) is the area under the PR curve, approximated by Eq. S7:

$$\mathrm{AP} = \sum_{k=1}^{n} (R_k - R_{k-1}) \times P_k \qquad \text{(Eq. S7)}$$

where P_k and R_k are the precision and recall at the k-th threshold, respectively, and n is the total number of thresholds. AP ranges from 0 to 1, with higher values indicating better overall performance. Unlike ROC-AUC, the PR curve is highly sensitive to class imbalance, making it more informative for highly imbalanced datasets where the positive class is rare.

A calibration curve (reliability curve) assesses whether a model's predicted probabilities reflect the true event frequencies. It is constructed using the binning method:

(1) Divide the predicted probability interval [0,1] into equally spaced bins.

(2) Assign each sample to a bin based on its predicted probability.

(3) For each bin, calculate the average predicted probability (x-axis) and the actual proportion of positive samples (y-axis).

(4) Plot the curve with average predicted probability on the x-axis and actual positive proportion on the y-axis.

The Brier score quantifies the accuracy of probability predictions and is defined as the mean squared difference between predicted probabilities and true outcomes (Eq. S8):

$$\text{Brier} = \frac{1}{N}\sum_{t=1}^{N}(f_t - o_t)^2 \qquad \text{(Eq. S8)}$$

where f_t is the predicted probability for the t-th sample, and o_t is the actual outcome (1 for positive cases, 0 for negative cases). The Brier score ranges from 0 to 1, with lower values indicating better calibration. It can be decomposed into three components—reliability, resolution, and uncertainty—for deeper diagnostic analysis.

Decision curve analysis (DCA) evaluates the decision utility of a predictive model. The core metric is Net Benefit, defined as Eq. S9:

$$\text{Net Benefit} = \frac{\text{TP}}{N} - \frac{\text{FP}}{N} \times \frac{P_t}{1 - P_t} \qquad \text{(Eq. S9)}$$

where N is the total sample size, TP is the number of true positives, FP is the number of false positives, and P_t is the given threshold probability. The term $P_t / (1 - P_t)$ reflects the relative weight of false positives. The DCA curve is constructed by calculating the net benefit over a range of threshold probabilities, along with the net benefits of two extreme strategies (Treat None and Treat All). The curve is plotted with threshold probability on the x-axis and net benefit on the y-axis. Among multiple models, the one with the highest net benefit across clinically relevant thresholds offers the best decision utility.

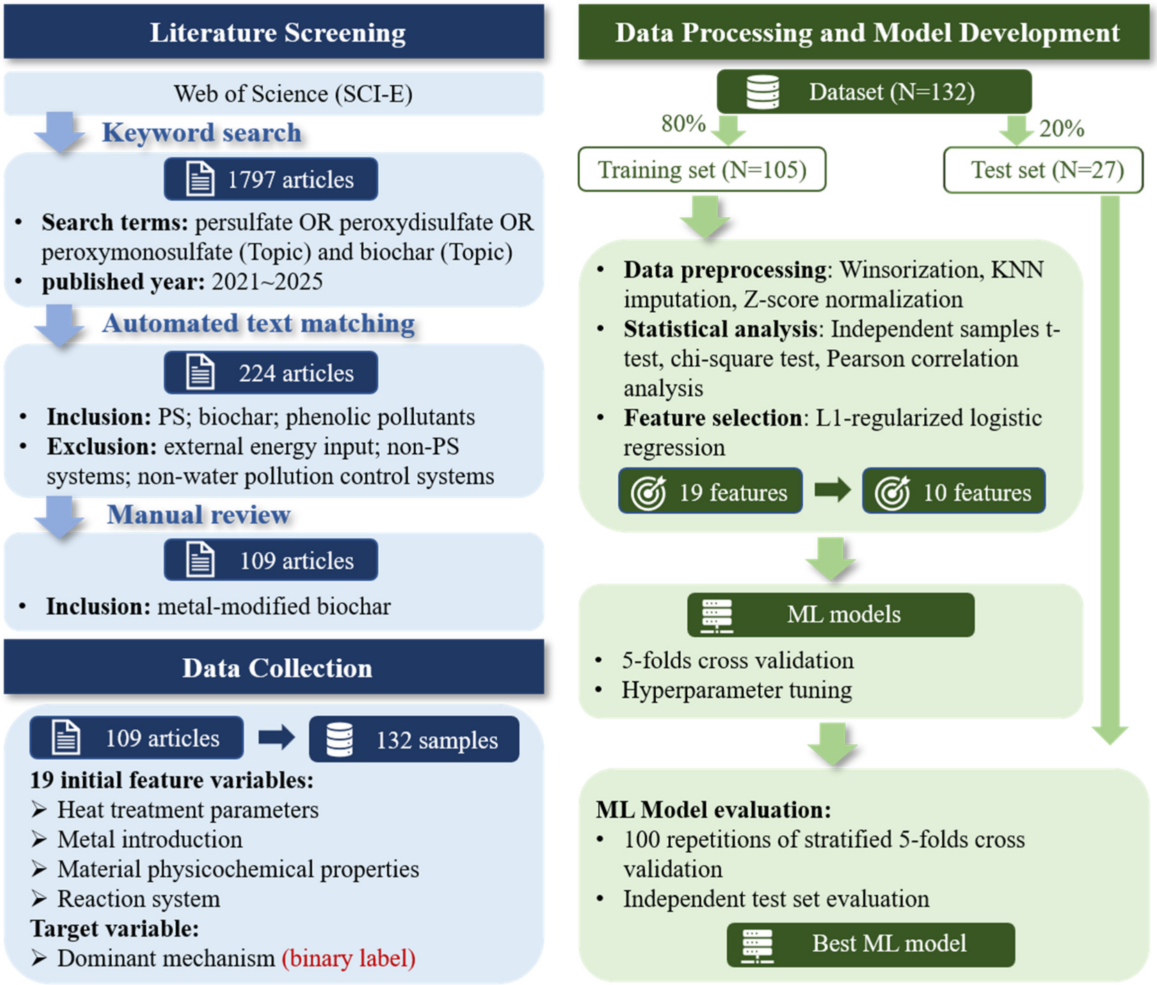

Figure S1. Comprehensive flowchart of the entire ML procedure.

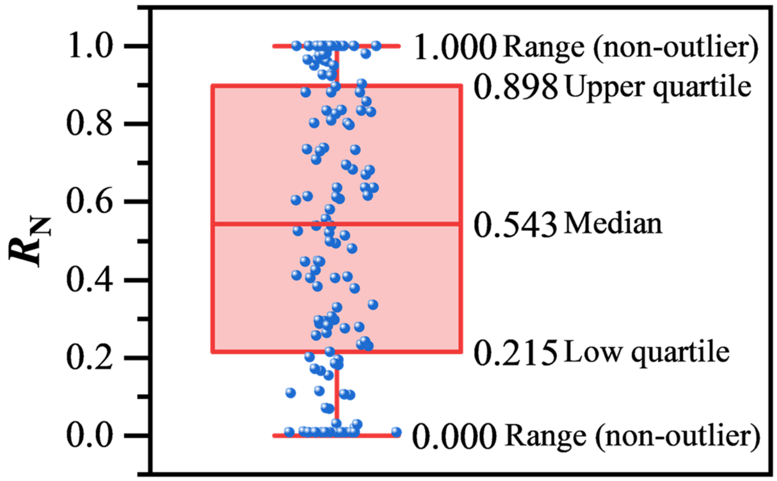

Figure S2. Boxplot of the contribution of non-radical pathways (R_N) in the full dataset.

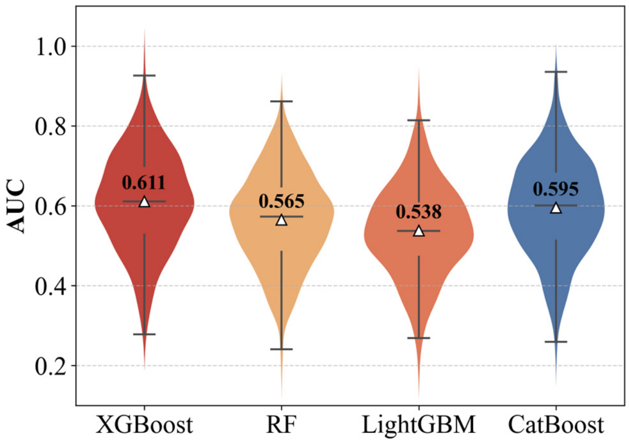

Figure S3. Distribution of AUC across 100 repetitions of stratified 5-CV for each model.

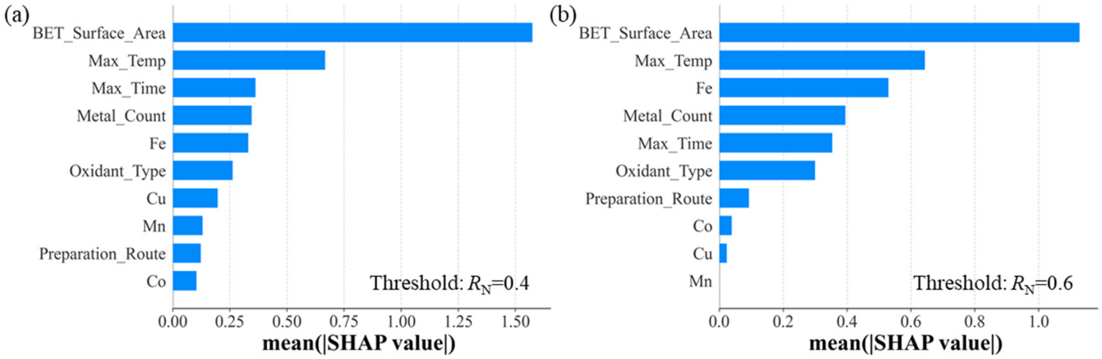

Figure S4. SHAP feature importance rankings under thresholds of (a) R_N = 0.4 and (b) R_N = 0.6.

Table S1. List of metal-modified biochar (MBC) catalysts and their corresponding Web of Science accession numbers (UT).

No.	MBC catalyst name	UT
1	nano-Fe/MC	WOS:000591554900017
2	ISA-Fe/MC	WOS:000591554900017
3	13%-Mn-FeBC	WOS:000624523300001
4	C800-1	WOS:000598929700007
5	C800-2	WOS:000598929700007
6	C800-3	WOS:000598929700007
7	Co_3O_4/C-BC	WOS:000669366700007
8	$CuFe_2O_4$/SBC	WOS:000647683300005
9	3SACu@NBC	WOS:000643902000001
10	nZVI/CuO@BC	WOS:000668981300004
11	G-nZVI-BC	WOS:000668980400001
12	CuO-Fe_3O_4-BC	WOS:000663209100006
13	nMnOx@RBC5	WOS:000692107800008
14	Fe_3O_4-BC	WOS:000687382700007
15	Ni-N-C-10	WOS:000682521000030
16	MCC	WOS:000711895600003
17	Fe(0)-included biochar	WOS:000701497200001
18	Biochar-coated Fe(0)	WOS:000701497200001
19	Fe_3S_4/BC	WOS:000743569600005
20	PWB-900	WOS:000740206500007
21	Fe/Mn@NBC800	WOS:000799083100001
22	BM-a-FeOOH/PBC700	WOS:000773839200002
23	PMC	WOS:000745198700001
24	Co/C-50	WOS:000764253200001
25	Co/C-50	WOS:000764253200001
26	Fe1-N-BC	WOS:000779659500001
27	Cu_0-Fe_3O_4@Biochar	WOS:000797598600001
28	nZVI@NBC	WOS:000791287000005
29	BC-Fe_0	WOS:000795095400003
30	$MnFe_2O_4$@BC	WOS:000867760100002
31	CuO/BC	WOS:000827382300005
32	Fe_3O_4/BC	WOS:000827382300005
33	ZnO/BC	WOS:000827382300005
34	Fe_3O_4@MBC800	WOS:000829219800002
35	F2BC3	WOS:000843372700006
36	α-MnO_2/0.1-CSB750	WOS:000861356500002
37	CMCC10%	WOS:000875853800004
38	Ni0.5/BC	WOS:000875818600001
39	Ni0.5/BC	WOS:000875818600001
40	Fe@N-BC400	WOS:000870578200009

Table S1 (continued). List of metal-modified biochar (MBC) catalysts and their corresponding Web of Science accession numbers (UT).

No.	MBC catalyst name	UT
41	Fe@N-BC600	WOS:000870578200009
42	Fe@N-BC800	WOS:000870578200009
43	F4@B5	WOS:000883244500003
44	F4@B7	WOS:000883244500003
45	F4@B9	WOS:000883244500003
46	FeS@NBCBM	WOS:000827643700002
47	FeBCG	WOS:000897763300005
48	MBC	WOS:000904166600008
49	PSBC-600	WOS:000906909900005
50	PSBC-800	WOS:000906909900005
51	PSBC-1000	WOS:000906909900005
52	nZVI0.3/NBC	WOS:000899382100001
53	ZVI@PSDBC	WOS:000923120400002
54	WFB20/Fe_3O_4	WOS:000938381600001
55	MBC/CuO	WOS:000938637900001
56	0.1Fe-800BC	WOS:000935375500001
57	nZVI0.6-BC800	WOS:000992855000001
58	Fe/Mn-BC	WOS:000946821500001
59	FeS/NBC	WOS:000965869400001
60	Cu-BC	WOS:001054044900001
61	Mg0.5Cu-BC	WOS:001054044900001
62	Mg1Cu-BC	WOS:001054044900001
63	Mg1.5Cu-BC	WOS:001054044900001
64	CuO/BC	WOS:000963793600001
65	Fe3C-BC	WOS:000994400300001
66	Fe/Mo0.1-NC	WOS:001000660400001
67	Fe-N-PGWBC	WOS:001003635600001
68	Fe_0@NC-0.8-0.6-800	WOS:001010706600001
69	Fe_0@NC-0.8-0-800	WOS:001010706600001
70	FeCu/OPB-1	WOS:001043777200001
71	Fe-CS@BC	WOS:001045954500001
72	BC@S-nZVI	WOS:001045844200001
73	Mn/C-KOH	WOS:001088505200001
74	$CuFe_2O_4$@CSBC	WOS:001100832600001
75	CSBC	WOS:001108250200001
76	Cu/HAP@sBC	WOS:001111268400001
77	BC@nZVI	WOS:001128952600001
78	CuO/BC-350	WOS:001130152500001
79	FeSA-BC	WOS:001171752800001
80	Mn@SBC-HP	WOS:001159873300002

Table S1 (continued). List of metal-modified biochar (MBC) catalysts and their corresponding Web of Science accession numbers (UT).

No.	MBC catalyst name	UT
81	Fe-ClBC	WOS:001198914600001
82	Fe-NBC	WOS:001198914600001
83	BC@FeMg-LDH	WOS:001173156100001
84	SACu30@NC	WOS:001217056500001
85	Mn@NBC-800	WOS:001224175100001
86	PBC/nZVI	WOS:001231082600001
87	Fe0.1-BDBC	WOS:001232044700001
88	nZVI@NBC	WOS:001241956600001
89	nZVI@Bi0/PPBC800	WOS:001243451200001
90	HC-2	WOS:001265954100001
91	Mn@NBC-800	WOS:001288201700001
92	Fe2Mn1@N-BC3-800	WOS:001289289300001
93	2% Cu3P/BPB	WOS:001364918200001
94	Cu-NBC	WOS:001355864200001
95	MnOx/CBC	WOS:001357471400001
96	CoMo@BC	WOS:001360076300001
97	(AB+MIL-88-0.5)-C	WOS:001366942400001
98	CoFe/BC	WOS:001376378500001
99	Fe-N-C	WOS:001382282200001
100	CoNBC600	WOS:001403576700001
101	ZVAl-BC700-FeATP	WOS:001413532900001
102	Fe/BC	WOS:001419864100001
103	Fe-N-C-700	WOS:001429857300001
104	FM@NBC-8	WOS:001441489400001
105	ZnS-NC/BC-1	WOS:001443427600001
106	P-Mn-TPs-900	WOS:001459407200001
107	FeCe0.05/BC	WOS:001463495900001
108	Fe_3O_4@BC	WOS:001482116300001
109	FeCo@NBC	WOS:001486634300003
110	Fe-CL/WT@BC	WOS:001501665600003
111	HC-DS-Al	WOS:001500149700003
112	V/BM-SD/BC	WOS:001509916900001
113	N/BM-SD/BC	WOS:001509916900001
114	A/BM-SD/BC	WOS:001509916900001
115	EMR-BC	WOS:001539263800001
116	Cu/C-HCl-HT	WOS:001536442100001
117	Fe1-xS/L-BC	WOS:001539862200002
118	CoP0.03/BC	WOS:001561381000001
119	S12F@BC	WOS:001573422300001
120	S12F@HBC	WOS:001573422300001

Table S1 (continued). List of metal-modified biochar (MBC) catalysts and their corresponding Web of Science accession numbers (UT).

No.	MBC catalyst name	UT
121	FeCu-N@OC500	WOS:001567305200001
122	CuNi-NC	WOS:001569440800001
123	FeN@BC	WOS:001576078600001
124	FKCB	WOS:001585845800003
125	A-FKCB	WOS:001585845800003
126	S-nZVI@BC	WOS:001593854700001
127	$CoMoO_4$@GLBC	WOS:001589337700002
128	1Cl-Fe-BC	WOS:001602955500004
129	Fe-S-SBC800	WOS:001606317000003
130	Fe-CBC	WOS:001605029400001
131	FVB700	WOS:001630836100003
132	$MnFe_2O_4$@PSBC	WOS:001649933700001

Table S2. Missingness and imputation methods of feature variables in the dataset.

Feature variable name	Missingness	Imputation method
Preparation_Route	0.000	/
Max_Temp	0.008	KNN*
Max_Time	0.015	KNN
Fe	0.000	/
Co	0.000	/
Ni	0.000	/
Cu	0.000	/
Mn	0.000	/
Al	0.000	/
Zn	0.000	/
Mg	0.000	/
Mo	0.000	/
Ce	0.000	/
Bi	0.000	/
Ca	0.000	/
Metal_Count	0.000	/
BET_Surface_Area	0.212	KNN
Id_Ig_Ratio	0.333	Excluded
Oxidant_Type	0.000	/
Non-radical	0.000	/

*KNN: K-nearest neighbor (K = 5).

Table S3. Average 5-CV AUC and optimal hyperparameters of each model.

Model algorithm	AUC	Optimal hyperparameters
XGBoost	0.699	{'learning_rate': 0.2, 'max_depth': 7, 'n_estimators': 50, 'subsample': 1.0}
LightGBM	0.653	{'learning_rate': 0.1, 'n_estimators': 200, 'num_leaves': 7, 'subsample': 0.6}
CatBoost	0.632	{'border_count': 64, 'depth': 10, 'iterations': 200, 'l2_leaf_reg': 1, 'learning_rate': 0.1}
RF	0.631	{'max_depth': 10, 'min_samples_split': 6, 'n_estimators': 200}

Table S4. Predictive performance of XGBoost model under various thresholds.

Threshold (R_N)	Training 5-CV AUC	Test AUC
0.4	0.708	0.665
0.5	0.699	0.682
0.6	0.617	0.698
